# Subcutaneous administration of adipose-tropic gene therapy for congenital leptin deficiency

**DOI:** 10.21203/rs.3.rs-7030870/v1

**Published:** 2025-07-17

**Authors:** Lei Cao, Wei Huang, Min Xiao, Xunchang Zou

**Affiliations:** Ohio State University; The Ohio State University

**Keywords:** AAV, adipose tropism, liver de-targeting, gene therapy, V7 capsid, leptin, adipose tissue, lipodystrophy, ob/ob mice, subcutaneous administration

## Abstract

AAV-based gene therapy targeting adipose tissue has been underdeveloped due to lack of adipose-tropic AAV vectors with sufficient transduction efficiency. We previously demonstrated that an engineered capsid variant of Rec2 capsid with F503Y, Y708D and K709I substitution (named V7 capsid) exhibited highly selective adipo-tropism while ablating liver transduction upon intraperitoneal injection or intravenous injection. In this study, we investigated the feasibility of subcutaneous administration of V7 vector harboring human leptin (V7-LEP) in a congenital leptin deficiency model *ob/ob* mice. Subcutaneous administration of V7-LEP vector at a low dose of 4×10^10^ viral genome per mouse restored circulating leptin levels and completely normalized metabolic abnormalities associated with leptin deficiency. In an ongoing long-term experiment, one-time subcutaneous administration of V7-LEP to extreme obese *ob/ob* mice has led to sustained weight loss at least up to 9 months post injection associated with stable circulating human leptin levels throughout the long-term study. These data indicate subcutaneous injection is a feasible and relevant administration route for gene therapy targeting adipose tissue, and V7-LEP is highly efficacious for congenital leptin deficiency and potentially other lipodystrophy disorders with leptin deficiency.

## INTRODUCTION

Recombinant Adeno-associated virus (AAV) vectors are not only valuable and versatile research tools for genetic manipulation *in vivo* but also the premier delivery system for gene therapy because of their non-pathogenic nature, broad tropism and long -term transgene expression in animal models and humans. Several AAV-based gene therapies have been approved by the US Food and Drug Administration or European Medicines Agency while AAV vectors have been evaluated in hundreds of clinical trials at various stages (1, 2, 3, 4). Although AAV vectors are efficient in delivering transgenes to liver, muscle, brain, and eye (5), its use in gene transfer to adipose tissue—one of largest organs in the body, remains limited largely due to low transduction efficiency and lack of adipose-tropism of natural AAV serotypes (6). As a result, adipose tissue, critically involved in physiology and pathologies of multiple diseases, is under studied in AAV-based gene therapy and strategies to improve adipose-tropism and/or restrict off-target transgene expression in non-adipose tissues are necessary to advance the field.

We previously reported that an engineered hybrid capsid Rec2, generated by capsid shuffling of natural serotypes AAV8 and rhesus 20 (rh20) (7), displays superior transduction efficiency to white adipose tissue (WAT) and brown adipose tissue (BAT) than the natural (AAV1, 8, and 9) and engineered capsids (Rec1, Rec3, Rec4) via direct intra-fat injection (8). However, Rec2 vector displays strong liver tropism upon systemic administration(9). To mitigate off-target transgene expression in the liver, we developed a first generation of adipose-targeting AAV vector by coupling Rec2 capsid with a dual-cassette AAV vector genome design which contains two expression cassettes: one uses constitutive hybrid cytomegalovirus enhancer/chicken β-actin (CBA) promoter to drive the transgene expression, and the other uses the liver-specific albumin promoter to drive a specific microRNA targeting woodchuck posttranscriptional regulatory element (WPRE) sequence within the transgene expression cassette and thereby silencing transgene expression in the liver. The Rec2/dual cassette vectors have achieved high efficacy in targeting adipose tissue in various mouse studies by our lab and collaborators (10, 11, 12, 13, 14, 15).

However, the Rec2/dual cassette has limitations such as inability to eliminating risk of viral vector-associated toxicity in liver at extremely high dose (16, 17), further exacerbating the size limit of a transgene, and not applicable to transgenes that are miRNA and shRNA. As such, we developed a second generation of adipose-targeting AAV vectors by site-specific mutagenesis on Rec2 capsid (18). We found the Rec2 capsid variant with F503Y, Y708D and K709I substitutions (referred to Rec2.V7, V7 in short) exclusively transduced adipose tissue while diminishing liver tropism upon systemic administration. Moreover, intraperitoneal (IP) injection of V7 capsid favors transduction to visceral fat while intravenous (IV) administration favors subcutaneous fat indicating administration route affects biodistribution of V7 capsid (18). Here, we characterized the V7 capsid via subcutaneous (SQ) injection—an administration route more relevant and desirable in clinical applications and conducted a proof-of-concept study mirroring previous study of V7 capsid via IP injection in the congenital leptin deficiency model *ob/ob* mice (18). Moreover, we are monitoring long-term therapeutic effects of V7 vector carrying human leptin gene (V7-LEP) in *ob*/*ob* mice up to date 9 months post a one-time SQ injection.

## MATERIALS AND METHODS

### Mice

Male homozygous *ob/ob* mice (JAX 000632 B6.Cg-Lep^ob^/J) and *ob*/+ mice were purchased from Jackson Laboratories. All mice were housed in temperature (22–23°C) and humidity (30–70%) controlled rooms under a 12:12 light:dark cycle. Mice were maintained on a standard chow diet (7912 rodent chow, Teklad), with *ad libitum* access to food and water. All use of animal was approved and in accordance with the Ohio State University Animal Care and Use Committee (IACUC), protocol approval 2012A00000060-R3.

### AAV vectors design and packaging

AAV trans-plasmid encoding rep and cap genes of adipose-tropic V7 capsid is described (18). The rAAV vector backbone contains CBA (hybrid cytomegalovirus–chicken β-actin) promoter, woodchuck hepatitis virus posttranscriptional regulatory element (WPRE) and bovine growth hormone polyadenylation signal flanked by AAV2-inverted terminal repeats. Reporter gene—enhanced green fluorescent protein (referred as GFP) or therapeutic gene—human leptin cDNA (referred as LEP, NCBI reference sequence: NM_000230.3) was cloned into polylinker sites of cis-plasmid as described previously (8). LEP sequence was synthesized by gBlock of Integrated DNA Technologies (IDT.) Cis-plasmids and trans-plasmid were prepared by using EndoFree plasmid Maxi Kit (Qiagen) and sent to vector packaging facilities. V7-GFP vector was packaged by VectorBuilder. V7-LEP was packaged by Forge Biologics.

### Administration of AAV vectors

*ob/ob* mice, four weeks old, were randomly assigned to receive V7-GFP or V7-LEP. Sex and age-matched heterozygous *ob/+* mice from the same colony served as lean control. Mice were anesthetized using rodent anesthesia (isoflurane) vaporizer chamber with 4% isoflurane in O_2_ for induction of anesthesia and 2–2.5% for maintaining it. Once anesthesia was fully induced, the animal was shaved with a hair trimmer over the area where inguinal WAT (iWAT) was anatomically located in groin area between the hindlimbs and the base of the tail. The injection was carried out around the base of the hindlimb. The animal was first laid flat on its stomach. Injection was performed without skin incision along with the orientation of iWAT using BD syringes needle (capacity:3/10 ml, length: 8 mm, Gauge: 31G). AAV vectors were administered in 25 μL AAV dilution buffer. Doses were 2×10^10^ vg each side, 4×10^10^ vg per mouse. All animals were included in data analysis.

### Body weight and food intake measurement

Body weight and food intake of *ob/ob* mice were monitored weekly over the period of 9 weeks. Food intake was measured at the cage level on a weekly basis throughout the experiment. Average food intake was calculated on a per-mouse per-day basis. Body weight and food intake of *ob*/+ mice were monitored from week 2 to week 9.

### Body composition by EchoMRI

EchoMRI was used to measure body composition of fat and lean masses in live mice without anesthesia at 4-weeks post AAV injection. EchoMRI imaging was performed with a 3-in-1 Analyzer (EchoMRI LLC, Houston, TX) according to manufacturer instructions at Small Animal Imaging Core of The Dorothy M. Davis Heart & Lung Research Institute, Ohio State University. Mice were subjected to a 5-Gauss magnetic field and whole-body masses of fat, lean, free water, and total water were determined during separate cycles by manufacturer software comparison to a canola oil standard.

### Glucose tolerance test (GTT)

Six weeks after administration of AAV vectors, mice were intraperitoneally injected with glucose solution (1mg glucose per gram body weight) after 16-h overnight fast with water *ad libitum*. Blood was collected via tail niche at 0, 15, 30, 60, 90, and 120 min after glucose injection and the blood glucose concentrations were measured with a portable glucose meter (Bayer Contour Next). Following blood collection, the styptic powder with Benzocaine (Kwik-Stop) was used to stop bleeding.

### Rectal temperature measurement

Seven weeks after AAV injection, rectal temperature was measured using a probe (Physitemp, model BAT-12) topped with lubricant (white petroleum jelly).

### Tissue collection

Mice were euthanized at 9-weeks post AAV injection after 4-h fast. Mice were anesthetized with 4% isoflurane (1.0 L/min) and then decapitated to collect trunk blood. Adipose tissues (interscapular brown adipose tissue, inguinal white adipose tissue, gonadal white adipose tissue, mesenteric white adipose tissue, anterior subcutaneous white adipose tissue, pericardial white adipose tissue), liver, heart, kidney, pancreas, spleen, and gastrocnemius muscle were collected and were flash frozen on dry ice and stored at −80°C until further analysis.

### Serum collection and analysis

Trunk blood was collected at euthanasia, clotted on ice, and centrifuged at 12,000 rpm for 15 min at 4°C. The serum was collected and stored at −20°C until further analysis. R&D Systems DuoSet ELISA kits were used to assay serum human leptin (R&D Systems, #DY398) and mouse leptin (R&D Systems, # DY498). Mouse serum insulin was measured with ELISA kit from ALPCO (ALPCO #80-INSMSU-E01). Caymen Chemical colorimetric assays were used to determine serum glucose levels (#10009582), alanine transaminase (ALT) levels (#700260), aspartate aminotransferase (AST) levels (#701640). The homeostatic model assessment for insulin resistance index (HOMA-IR) was calculated as [fastingserumglucose(mmol/L)×fastingseruminsulin(pmol/L)/22.5].

### Liver lipid extraction and triglycerides assay

Hepatic lipid was extracted from 15–30 mg of wet liver tissue from each animal by chloroform/methanol (2:1 v/v), followed by rinse in 50 mM NaCl and CaCl_2_ (0.36M)/Methanol (1:1 v/v)(9). An aliquot of the extract was mixed with Triton X100 and cold acetone, then was dried up and redissolved in PBS. Hepatic triglyceride quantification was performed with a Caymen Chemical triglyceride assay kit (#10010303).

### Quantitative RT-PCR

Total RNA was isolated using the RNeasy Mini kit (QIAGEN) with RNase-free DNase treatment. cDNA was reverse transcribed using Taqman Reverse Transcription Reagents (Applied Biosystems). qPCR was carried out on StepOnePlus Real-Time PCR System using Power SYBR Green PCR Master Mix (Applied Biosystems). Primer sequences are available in Table 1. Data were calibrated to housekeeping controls *Actb* for adipose tissues and *Ppia* for liver. The relative gene expression was quantified using the 2^− ΔΔCT^ method.

### AAV vector copy number measurement

To assess vector biodistribution, viral vector copy number was measured as previously described(10). Total DNA from various tissues was isolated using DNeasy Blood and Tissue kit (Qiagen). WPRE fragment was amplified and quantified by qPCR to determine the copy number of viral particle. 50 ng of DNA from each sample was used for real-time PCR with duplicate. Mouse nucleic genomic DNA fragment of BDNF gene was used as control for mouse genetic DNA. Genomic DNA of untreated animals was also used as negative control for the comparison. Data are expressed as viral genome copies per 50 ng of genomic DNA.

### Long-term experiment

Male *ob/ob* mice, 17 weeks old with body weight between 57 and 62 grams, received V7-LEP either via SQ injection (two mice) or IP injection (one mouse). The dose was 1×10^11^ vg per mouse. Body weight is monitored weekly. Serum human leptin levels are measured by ELISA (R&D Systems, #DY398) at 2.7-, 17-, 27-, and 32-weeks post V7-LEP injection. The long-term experiment is in progress.

### Qualification and Statistical analysis

Data are expressed as means ± SEM. GraphPad Prism 10 was used to analyze data. One-way ANOVAs with Tukey’s *post hoc* test was utilized for comparisons between three groups. Time course data (BWs, GTT) were analyzed using a mixed ANOVA and area under the curve calculations were performed where applicable. Normality was tested using the Shapiro-Wilk method. * Indicates P value < 0.05; ** indicates P value < 0.01; *** indicates P value < 0.001; **** indicates P value < 0.0001 in figures.

## RESULTS

### Subcutaneous administration of V7-LEP rescues the excessive weight gain, hyperphagia, abnormal body temperature, and impaired glycemic control in ob/ob mice.

To investigate the efficacy of SQ injection, we conducted an experiment mirroring previously published studies using the same *ob/ob* mouse model, dose of adipose-targeting AAV vector expressing leptin, and *in vivo* metabolic assessments in which IP administration were used (10, 18). In rodents, fat depots are distributed in various locations—subcutaneous depots include intrascapular brown adipose tissue (BAT), inguinal white adipose tissue (iWAT), and anterior white adipose tissue (aWAT); visceral or intraperitoneal fat depots include epididymal (male) or gonadal (female) white adipose tissue (eWAT or gWAT), mesentery white adipose tissue (mWAT), and retroperitoneal white adipose tissue (rWAT). Male *ob/ob* mice, 4 weeks of age, were randomized to receive SQ administration of V7-LEP or V7-GFP adjacent to the iWAT (2×10^10^ vg each side, 4×10^10^ vg/mouse). Age-matched male *ob* heterozygote (*ob*/+) from the colony was used as a lean control for normal metabolic phenotypes.

**Figure 1:**
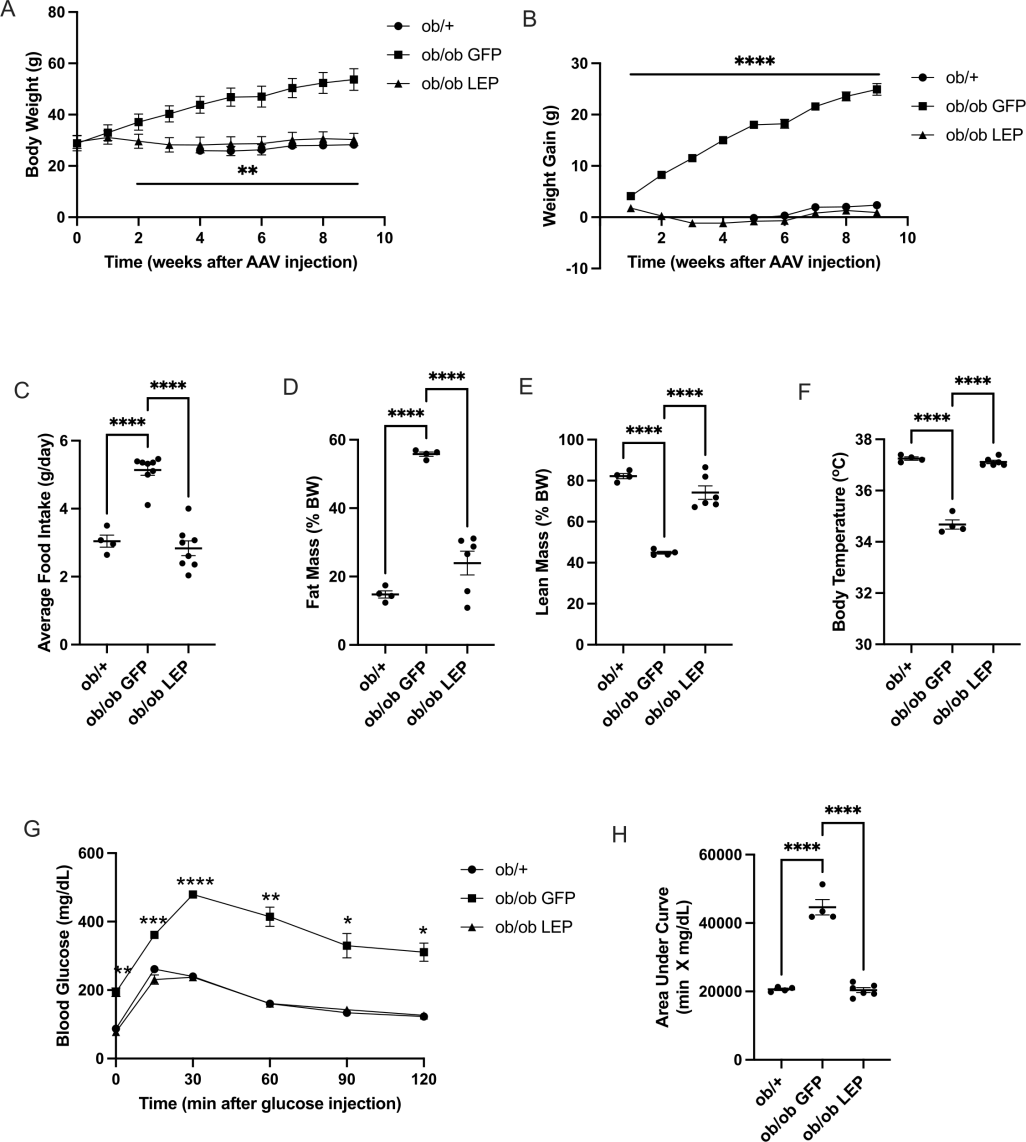
Subcutaneous administration of V7-LEP rescues the excessive weight gain, hyperphagia, abnormal body temperature, and impaired glycemic control in *ob/ob* mice. (**A**) Body weight. (**B**) Weight gain from baseline. (**C**) Average food intake from week 1 to week 9 post AAV injection for *ob/ob*GFP and *ob/ob* LEP; from week 2 to week 9 for *ob*/+ group. (**D**) Relative fat mass (**E**) Relative lean mass at 4-weeks post AAV injection. (**F**) Core temperature at 7-weeks post AAV injection. (**G**) Glucose tolerance test at 6-weeks post AAV injection. (**H**) Area under the curve of glucose tolerance. * *P*<0.05, ** *P*<0.01, ***P<0.001, ****P<0.0001 *ob*/*ob* GFP vs all other groups. No significant difference *ob*/+ vs *ob/ob* LEP. Data are mean±SEM. n=4 *ob*/+, n=4 *ob/ob* GFP, n=6 *ob/ob* LEP.

*ob/ob* mice receiving V7-GFP displayed rapid weight gain over the 9 weeks period of the experiment with a total weight gain reaching 24.9 ± 1.1 g, approximately 10-folds higher than *ob*/+ mice (Fig. 1A, 1B). In contrast, the weight of *ob/ob* mice receiving V7-LEP was significantly lower than V7-GFP injected *ob/ob* mice starting at two weeks after SQ injection, and the body weight was normalized to the level of *ob*/+ mice (Fig. 1A, 1B). The total weight gain of *ob/ob* mice treated with V7-LEP was 0.92 ± 0.48 g over 9 weeks, 20-folds lower than *ob/ob* mice treated with V7-GFP. V7-LEP treatment completely reversed hyperphagia (Fig. 1C) throughout the duration of the study.

### Subcutaneous administration of V7-LEP normalizes adipose tissue mass and lean tissue mass

At 4 weeks post AAV injection, body composition was determined by echoMRI. V7-LEP treatment reduced adiposity and increased lean mass in *ob/ob* mice to the levels in lean *ob*/+ control (Fig. 1D, 1E). A glucose tolerance test was performed at 6 weeks post AAV injection. V7-LEP treatment completely normalized the fasting blood glucose level and glucose tolerance (Fig. 1G, 1H). As *ob/ob* mice exhibit abnormal low body temperature due to downward shifted thermoregulatory thresholds (19), core body temperature was measured at 7 weeks post AAV injection. V7-LEP treatment fully corrected the abnormal low body temperature of *ob/ob* mice (Fig. 1F).

**Figure 2:**
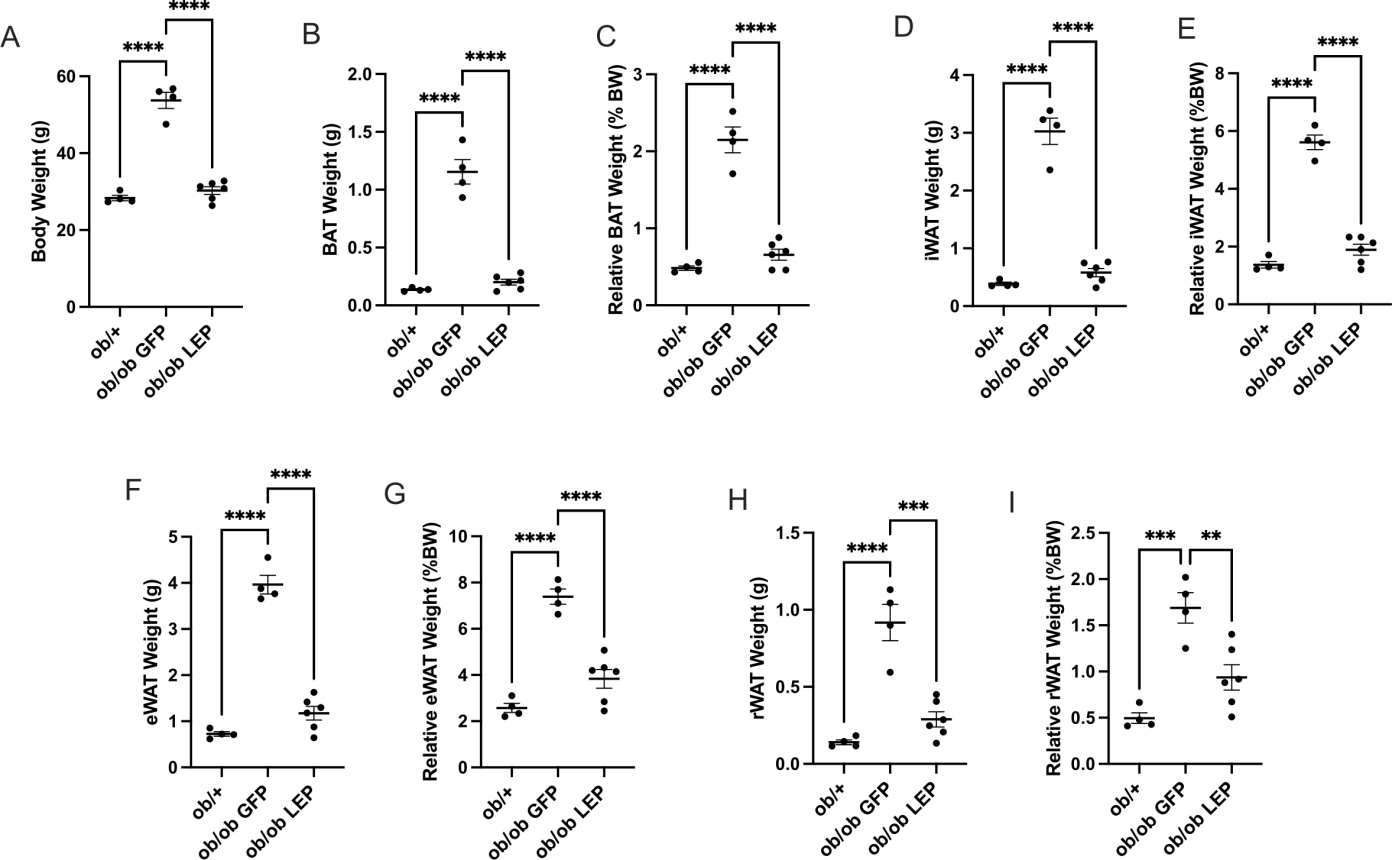
Subcutaneous administration of V7-LEP normalizes adipose tissue mass. (**A**) Final body weight. (**B**) Brown adipose tissue (BAT) mass. (**C**) Relative BAT mass. (**D**) Inguinal white adipose tissue (iWAT) mass. (**E**) Relative iWAT mass. (**F**) Epididymal white adipose tissue (eWAT) mass. (**G**) Relative eWAT mass. (**H**) Retroperitoneal white adipose tissue (rWAT) mass. (**I**) Relative rWAT mass. Data are means±SEM. Sample size (biological replicates): n=4 *ob*/+, n=4 *ob/ob*GFP, n=6 *ob/ob* LEP. * *P*<0.05, ** *P*<0.01, ***P<0.001, ****P<0.0001.

Consistent with echoMRI data, at euthanasia 9 weeks after V7 vectors administration, the weight of all collected adipose depots, including BAT, eWAT, iWAT, and rWAT in V7-LEP treated *ob/ob* mice were all normalized to the levels observed in *ob*/+ mice (Fig. 2B, 2D, 2F, 2H). Compared to *ob/ob* mice treated with V7-GFP, the relative mass of fat depots in V7-LEP mice were reduced ranging from 50–70% (Fig. 2C, 2E, 2G, 2I).

**Figure 3:**
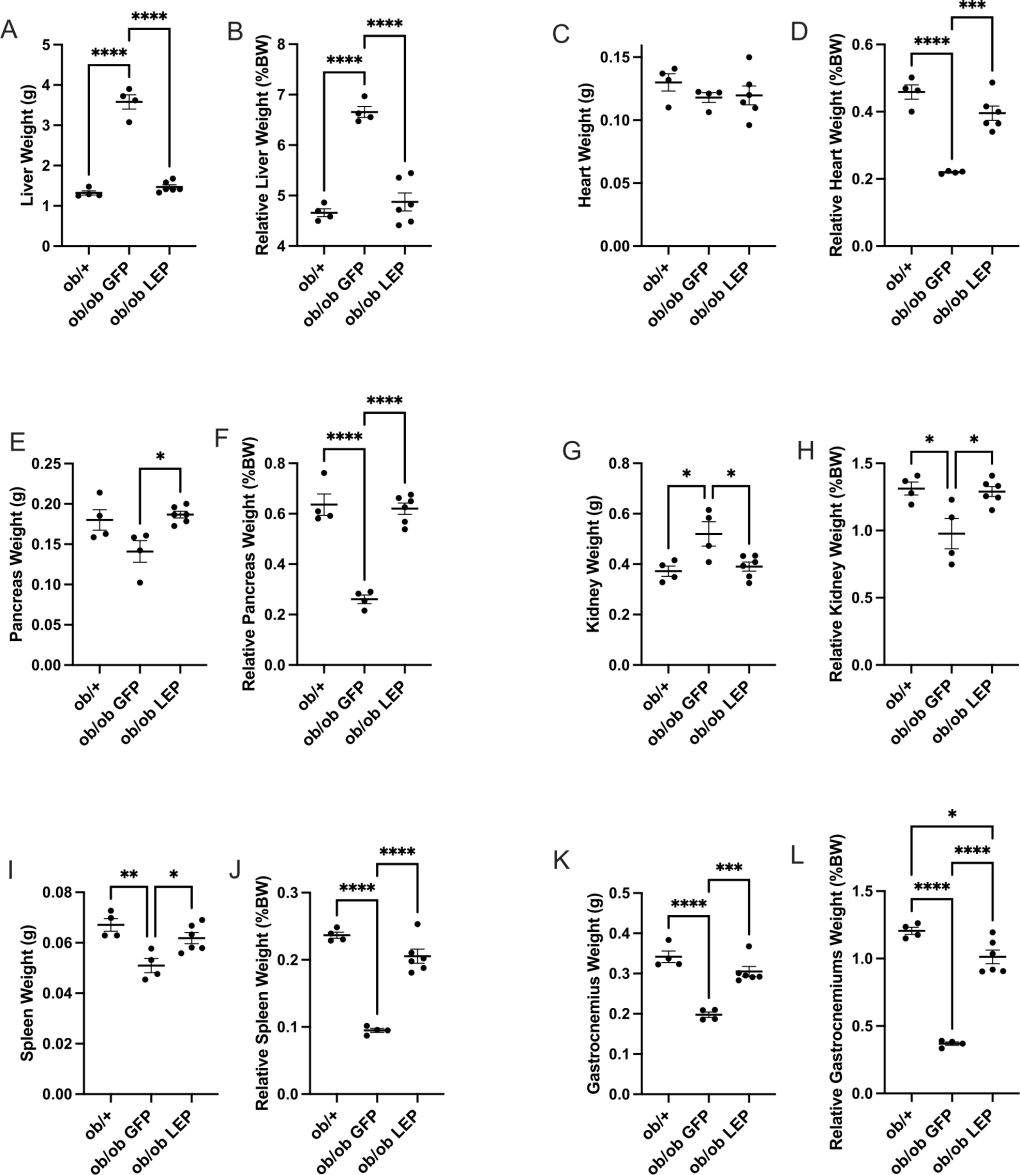
Subcutaneous administration of V7-LEP normalizes lean tissue mass. (**A**) Liver weight. (**B**) Relative liver weight. (**C**) Heart weight. (**D**) Relative heart weight. (**E**) Pancreas weight. (**F**) Relative pancreas weight. (**G**) Kidney weight. (**H**) Relative kidney weight. (**I**) Spleen weight. (**J**) Relative spleen weight. (**K**) Gastrocnemius weight. (**L**) Relative gastrocnemius weight. Data are means±SEM. Sample size (biological replicates): n=4 *ob*/+, n=4 *ob/ob*GFP, n=6 *ob/ob* LEP. * *P*<0.05, ** *P*<0.01, ***P<0.001, ****P<0.0001.

*ob* genotype-associated changes in nonfat tissue mass including liver (Fig. 3A, 3B), heart (Fig. 3C, 3D), pancreas (Fig. 3E, 3F), kidney (Fig. 3G, H), spleen (Fig. 3I, 3J) and gastrocnemius muscle (Fig. 3K, 3L) were normalized by V7-LEP treatment.

### Subcutaneous administration of V7-LEP restores leptin level and reverses hyperinsulinemia, insulin resistance, and hepatic steatosis in ob/ob mice

**Figure 4:**
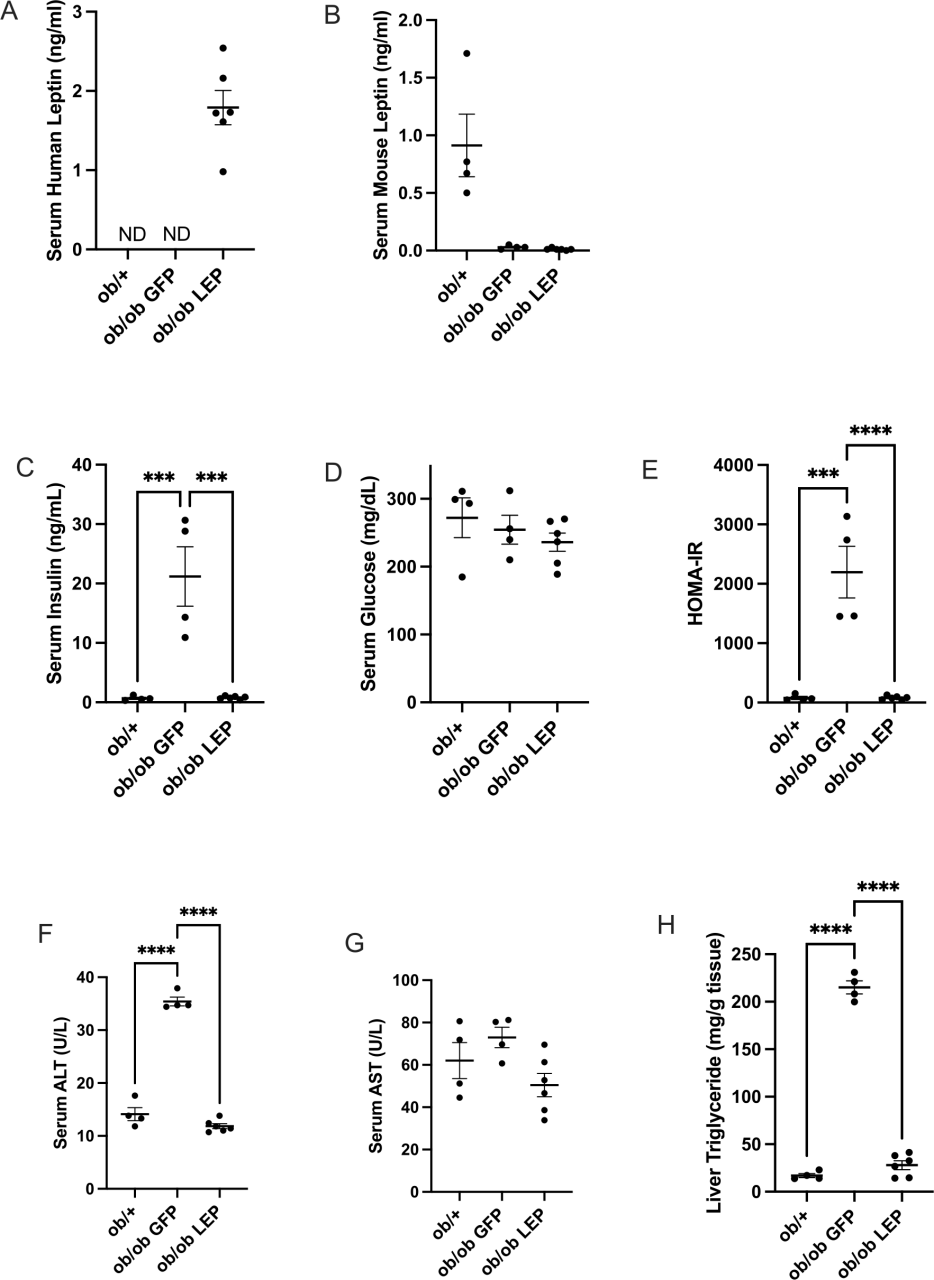
Subcutaneous administration of V7-LEP restores leptin level and reverses hyperinsulinemia, insulin resistance, and hepatic steatosis in *ob/ob* mice. (**A**) Serum human leptin. ND, not detectable. (**B**) Serum mouse leptin. (**C**) Serum insulin. (**D**) Serum glucose. (**E**) Homeostatic model assessment for insulin resistance index (HOMA-IR). (**F**) Serum Alanine transaminase (ALT). (**G**) Serum Aspartate transferase (AST). (**H**) Liver triglyceride content. Data are means±SEM. Sample size (biological replicates): n=4 *ob*/+, n=4 *ob/ob*GFP, n=6 *ob/ob* LEP. * *P*<0.05, ** *P*<0.01, ***P<0.001, ****P<0.0001.

Serum levels of human leptin derived from V7-LEP vector were measured with an ELISA without cross reaction to mouse counterparts. The serum levels of human leptin and adiponectin showed a dose dependent effect although not reaching statistical significance (Fig. 6A). Human leptin level in V7-LEP mice was 1.79 ± 0.22 ng/ml while undatable in *ob*/+ mice and V7-GFP mice (Fig. 4A). The human leptin level in V7-LEP mice exceeded the serum level of mouse leptin in the *ob*/+ control mice (0.91 ± 0.27 ng/ml, Fig. 4B). The *ob*-genotype associated extreme hyperinsulinemia was completely reversed by V7-LEP treatment with a 27-fold reduction in serum insulin level compared to V7-GFP mice (Fig. 4C). Moreover, V7-LEP treatment completely normalized the homeostatic model assessment for insulin resistance index (HOMA-IR) to the level of *ob* heterozygote mice (Fig. 4E). V7-LEP treatment reversed the elevated level of alanine transaminase (ALT) in *ob/ob* mice (Fig. 4F) while no genotype- or gene therapy-associated change in serum glucose (Fig. 4D) or aspartate aminotransferase (AST) (Fig. 4G) were observed. Hepatic triglyceride content in V7-LEP mice was reduced 87% compared to V7-GFP mice, reaching a comparable level of *ob* heterozygote mice (Fig. 4H), indicating reversal of liver steatosis in *ob/ob* mice.

### Subcutaneous administration of V7-LEP mitigates leptin deficiency-driven gene expression signature in the liver and white adipose tissue.

**Figure 5:**
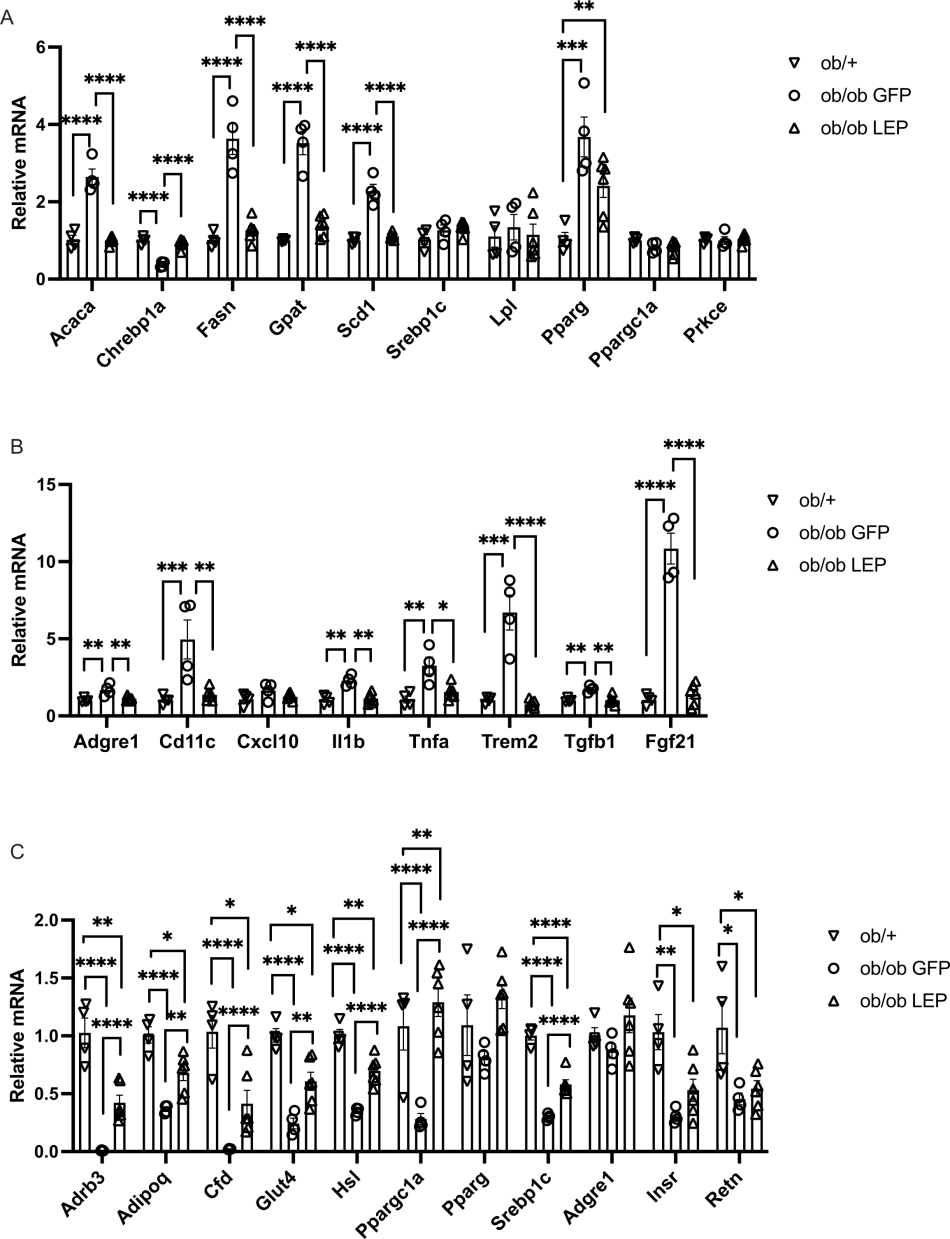
Subcutaneous administration of V7-LEP mitigates leptin deficiency-driven gene expression signature in the liver and white adipose tissue. (**A**) and (**B**) Gene expression profile of liver. (**C**) Gene expression profile of inguinal white adipose tissue. Data are means±SEM. Sample size (biological replicates): n=4 *ob*/+, n=4 *ob/ob* GFP, n=6 *ob/ob* LEP. ***P<0.001, ****P<0.0001.

Hepatic gene expression was profiled by qRT-PCR. Genotype-driven changes in genes involved in hepatosteatosis were observed including *Acaca* (encoding acetyl-CoA carboxylase alpha, ACC1), *Chrebp1a* (encoding carbohydrate-responsive element-binding protein isoform a), *Fasn* (encoding fatty acid synthase), *Gpat* (encoding glycerol-3-phsphate acyltransferase), *Scd1* (encoding stearoyl-coenzyme A desaturase 2), and *Pparg* (encoding peroxisome proliferator-activated receptor gamma). These genotypic changes were all reversed by V7-LEP treatment (Fig. 5A). No genotype- or gene therapy-associated changes were observed in the expression of *Srebp1c* (encoding sterol regulatory element-binding protein 1, isoform c), *Lpl* (encoding lipoprotein lipase), *Ppargc1a* (encoding peroxisome proliferator-activated receptor gamma coactivator 1-alpha) or *Prkce* (encoding Protein kinase Cε) (Fig. 5A).

In addition, genotype-driven upregulation of macrophage markers and inflammation related genes was observed, including *Adgre1* (encoding adhesion G protein-coupled receptor E1, also known as F4/80), *Cd11c* (encoding integrin alpha X), *Il1b* (encoding interleukin 1 beta), *Tnfa* (encoding tumor necrosis factor-α), *Trem2* (encoding triggering receptor expressed on myeloid cells 2), and *Tgf1* (encoding transforming growth factor beta 1). V7-LEP treatment reversed this hepatic proinflammatory gene signature driven by leptin deficiency (Fig. 5B). *ob/ob* mice share many features with lipodystrophy although being obese (20, 21). Lipodystrophy is associated with upregulation of *Fgf21* (encoding fibroblast growth factor 21) (22). A 10-fold genotype-driven upregulation of *Fgf21*was found in the liver of *ob/ob* GFP mice, which was normalized by V7-LEP treatment (Fig. 5B).

In the iWAT, a genotype-driven gene signature characterized as robust downregulation of adipose functional genes was revealed, including *Adrb3* (encoding adrenoceptor β3) mediating sympathetic regulation; major adipokines such as *Adipoq* (encoding adiponectin), *Cfd* (encoding complement factor D, or adipsin), and Retn (encoding resistin); *Insr* (encoding insulin receptor) and *Glut4* (encoding glucose transporter 4) critical for glucose metabolism; *Hsl* (encoding hormone-sensitive lipase), crucial enzyme in lipolysis; Srebp1c, transcription factor regulating lipgenesis; *Ppargc1a*, a transcriptional coactivator regulating genes involved in energy metabolism (Fig. 5C). V7-LEP treatment restored the expression of *Ppargc1a* and significantly mitigated genotype-driven downregulation of *Adrb3*, *Adipoq*, *Cfd*, *Hsl*, and *Srebp1c* (Fig. 5C).

### V7-LEP biodistribution

**Figure 6:**
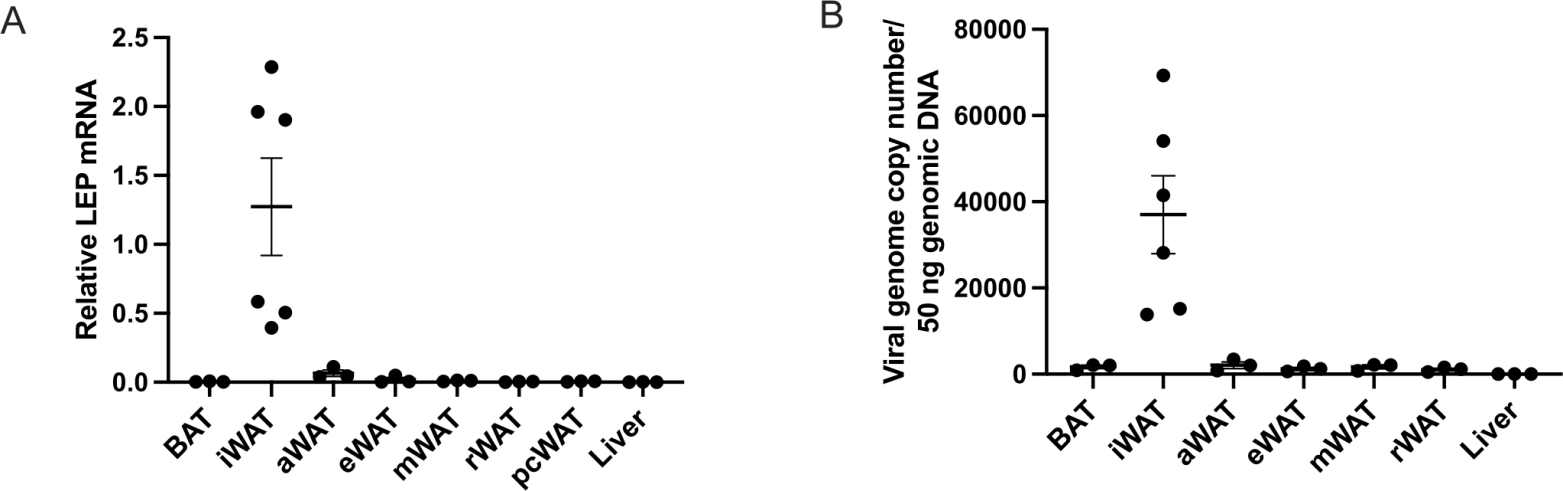
V7-LEP biodistribution. (**A**) LEP mRNA. (**B**) Viral genomes copy numbers. Data are means±SEM. Sample size (biological replicates): n=6, iWAT, n=3 other tissues. BAT, brown adipose tissue; iWAT, inguinal white adipose tissue; aWAT, anterior white adipose tissue; eWAT, epididymal white adipose tissue; mWAT, mesenteric white adipose tissue; rWAT, retroperitoneal white adipose tissue; pcWAT, pericardial white adipose tissue.

We previously reported that V7 vectors via IP or IV injections at the dose up to 8×10^10^ vg per mouse led to selective transduction of adipose tissue while diminished transduction of liver and other major peripheral organs (heart, pancreas, kidney, spleen, muscle, lung, intestine) and brain (18). To validate adipose-tropism of V7-LEP via SQ administration, transgene mRNA and vector genome were determined in various adipose depots and liver. The primers were designed to detect human LEP transgene without cross reaction to mouse *Lep*. Human LEP transgene expression was mainly detected in the iWAT where the SQ injection occurred while low level transgene mRNA detected in a distant subcutaneous depot anterior WAT (aWAT) but not in the liver (Fig. 6A). Vector genome distribution was consistent with the transgene mRNA data, highly enriched in the iWAT while minimally detected in visceral adipose depots or liver (Fig. 6B).

### Long-term effects of V7-LEP in obese ob/ob mice

**Figure 7:**
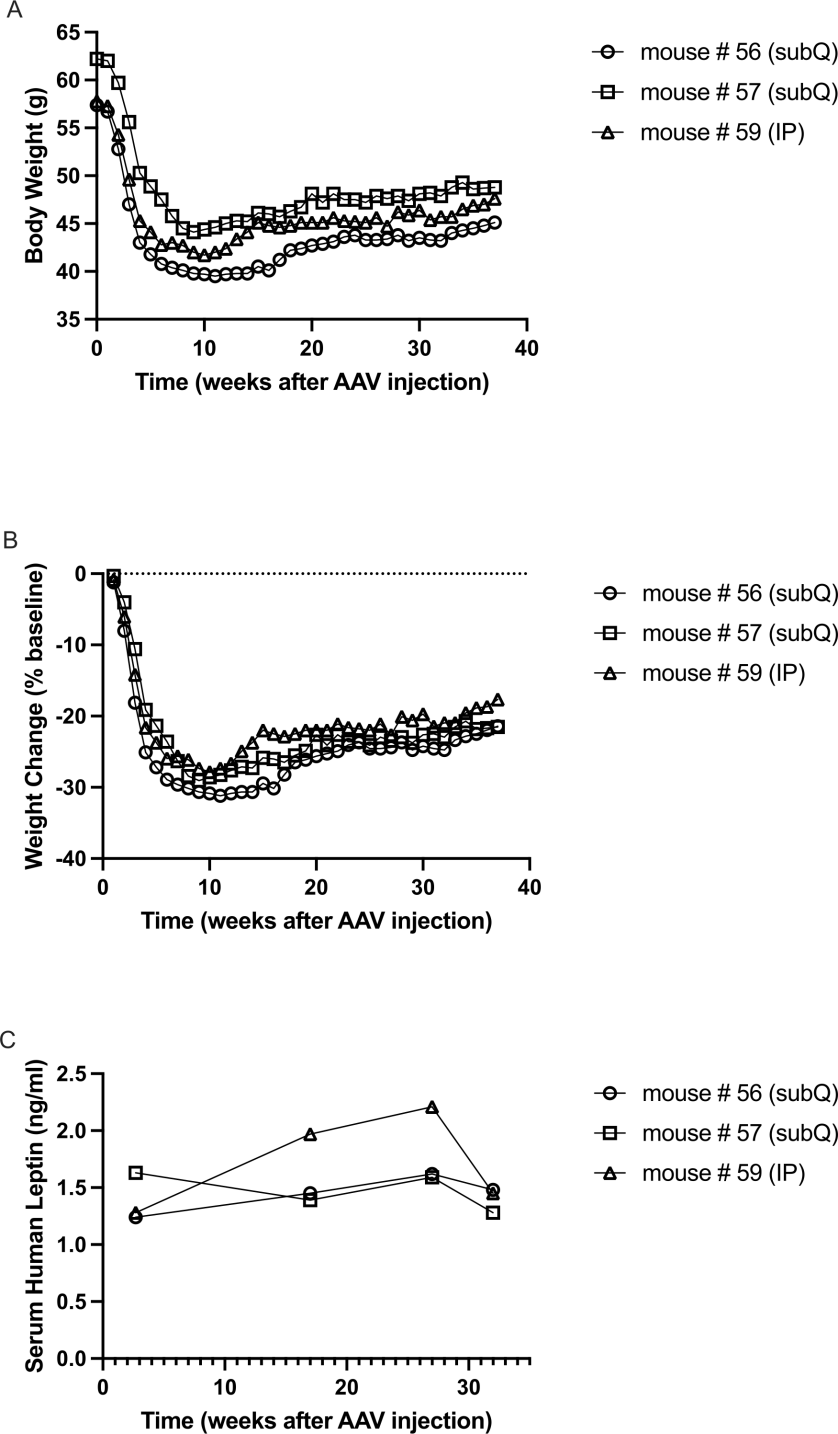
Long-term effects of V7-LEP in obese *ob/ob* mice. (**A**) Body weight. (**B**) Weight change from baseline. (**C**) Serum human leptin levels at 19-days, 17-weeks, 27-weeks, and 32-weeks post AAV injection. Data are individual animal receiving V7-LEP (1×10^11^ vg per mouse), Mouse #56 and #57 via subcutaneous injection, Mouse #59 via intraperitoneal injection.

Evidence on whether leptin replacement is effective when extreme obesity develops in the *ob/ob* mice remains scarce. We conducted a pilot study in 17-weeks old *ob/ob* mice when the body weight was between 57 and 62 grams, more than 2-folds of the weight of wildtype littermate. Two mice received SQ injection of V7-LEP and one mouse received IP injection of V7-LEP, all at the dose of 1×10^11^ vg per mouse. V7-LEP treatment led to approximately 30% weight loss 6–10 weeks post injection and > 20% weight loss is sustained up to 37 weeks post injection (Fig. 7A, 7B). Serum levels of human leptin were measured periodically at 19-days, 17-, 27-, and 32-weeks post V7-LEP injection. Circulating human leptin levels are sustained up to 8 months post an one-time injection regardless the administration route (SQ or IP). The long-term experiment is in progress with weekly weight recording and monthly serum human leptin measurement going forward.

## DISCUSSION

Adipose-targeting AAV vectors unrelated to capsid modification often combine adipocyte-specific promoter and the targeting sequences of endogenous microRNAs that selectively expressed in the tissues where transgene expression is to be avoided. Most studies report doses of systemic administration exceeding 1×10^12^ vg per mouse (6, 23, 24, 25). The V7 capsid with three residue substitutions (F503Y, Y708D and K709I) on Rec2 capsid represents the first of a new class of engineered AAV capsids that transduce adipose tissue exclusively while de-targeting liver and other tissues by systemic administration (18). The improved adipose tropism of V7 capsid enables the use of nonrestrictive expression cassettes to achieve adipose-specific transgene expression at lower doses and thereby reducing the cost of AAV-based gene therapy and viral vector-associated toxicity.

The main objective of this study was to establish SQ delivery as a feasible administration route. SQ administration is more relevant to clinical use in humans than rodent models due to the distinct anatomical distribution of adipose tissues between the two species. In humans, SQ injection often delivers therapeutic agents to fatty tissue layer beneath the skin and therefore is likely the favorable administration route for adipose-targeting gene therapy. In contrast, the main SQ depots in mice are not as abundant as in humans, thus SQ injection in mice is unlikely to deliver all agents to the subcutaneous fat depots. As such, the gene delivery efficiency of V7 vector revealed in murine models might underestimate its efficacy in species whose fat distribution more like humans. Here, we demonstrate that SQ injection of V7-LEP at a low dose of 4×10^10^ vg per mouse restored circulating leptin levels comparable to IP injection of V7 vector (18) or IP injection of Rec2/dual cassette vector (10) and normalized the metabolic syndromes in *ob/ob* mice. These results indicate SQ administration is feasible for systemic gene therapy in which therapeutic molecules are produced in adipose tissue and act on targets other than adipose tissue. Furthermore, a low dose of V7-LEP at 4×10^10^ vg per mouse achieved circulating human leptin exceeding the mouse leptin level of *ob* heterozygous mice. As restoring the circulating leptin level to 10% of normal levels is sufficient to alleviate the metabolic syndromes associated with congenital leptin deficiency (23), the dose of V7-LEP via SQ administration could be further reduced. We recently conducted a dose-deescalating study in *ob/ob* mice to find the minimal effective dose for the Rec2/dual cassette vector expressing mouse leptin. IP injection of Rec2 based liver-restricting leptin vector at doses as low as 1×10^9^ vg per mouse was sufficient to rescue leptin deficiency and associated metabolic syndrome (26). Future study will assess if V7 capsid based gene therapy allows a dose, hundreds-times lower than commonly reported doses for systemic AAV-based gene therapy. Moreover, literature on SQ administration of AAV vectors remains limited (27, 28, 29). The efficacy data from this preclinical study will encourage efforts on SQ as a feasible and more translatable administration route for gene therapy.

Biodistribution assessment confirmed the adipose tropism and liver de-targeting property of the V7 capsid. Pattern of fat depots to be transduced depends on the administration routes: IP administration favoring visceral fat depots (18); IV administration favoring subcutaneous WAT and BAT (18); SQ largely restricting to the subcutaneous fat adjacent to injection (Fig. 6). Of note, low level of transgene mRNA was detected in the aWAT distant from the injection site near iWAT suggesting transduction via circulation. Given a low dose of 2×10^10^ vg per injection (4×10^10^ vg per mouse) was used in the study, it is plausible that higher doses could result in more widespread transduction of fat depots. Nevertheless, IP, SQ, or IV administrations can be combined in case whole body adipose gene transfer is desirable.

Congenital leptin deficiency is an ultrarare genetic disease derived from monogenic defect in adipose tissue (30), causing hyperphagia, severe obesity, and hyperinsulinemia, which is associated with high morbidity and mortality if untreated (31). Moreover, leptin deficiency shares many symptoms with lipodystrophy, a group of heterogeneous disorders, inherited or acquired, characterized by loss of functional adipose tissue (20, 21). Currently, leptin replacement therapy with Metreleptin, a recombinant leptin protein analog, is the only specific therapy for congenital leptin deficiency or lipodystrophy syndrome (32, 33, 34). Metreleptin therapy requires frequent SQ injection for life, and can cost $1.3 million per patient per year (https://www.drugs.com/article/top-10-most-expensive-drugs.html). Moreover, serious side effects can occur possibly due to the supra-physiological surge of circulating leptin level following regular injection (35). As such, gene therapy based on an adipo-tropic AAV vector may offer significant advantages compared to current standard of care. This preclinical study demonstrates that V7-LEP via SQ administration at a low dose of 4×10^10^ vg per mouse completely normalized leptin deficiency and associated metabolic syndromes, supporting translational potential.

Profiling of hepatic and adipose gene expression in leptin replacement remains limited (36, 37). This study provides new dataset not available in previous studies of leptin gene therapy in *ob/ob* mice (10, 18, 23). V7-LEP treatment led to a complete reversal of genotype-driven gene expression signature in the liver of *ob/ob* mice and partial mitigation of the molecular features in WAT driven by leptin deficiency (Fig. 5). Whether the V7-LEP gene therapy can be generalized to other lipodystrophy conditions warrant investigation. We are testing the V7-LEP gene therapy in a congenital generalized lipodystrophy model, aP2-SREBP1c transgenic mice that overexpress human nuclear sterol regulatory element-binding protein-1c (nSREBP-1c/ADD1) specifically in adipose tissue (38).

In our previous studies (10, 18), leptin vectors, either Rec2 vector or V7 vector, were administered at earlier age, 4–6 weeks, as most leptin replacement studies were conducted (23). Here, we demonstrate that V7-LEP administered after occurrence of extreme obesity (57–63 g) results in 20–30% weight loss. The weight loss and circulating human leptin levels have been stable and sustained at least 9 months post systemic administration (SQ or IP). The durability data in this long-term study has exceeded all our previous studies using adipose-targeting AAV vectors (8, 10, 11, 12, 13, 39, 40). This in progress study continues until substantial drop of circulating human leptin is observed. Our adipo-tropic AAV platform has the potential to enabling lower overall systemic doses to patients, resulting in fewer toxicities and much lower cost of goods for systemically delivered gene therapies.

In summary, our data demonstrate that subcutaneous administration of an adipose-tropic AAV capsid vector restored circulating leptin level and normalized metabolic abnormalities in a murine model of congenital leptin deficiency at a dose substantially lower than standard AAV systemic gene therapy. One-time subcutaneous administration of the V7-LEP led to stable and sustained transgene expression and therapeutic effect at least up to 9 months after injection. These data establish subcutaneous delivery as a feasible administration route for adipose-targeting gene transfer. Further development of the V7 vector platform will provide a powerful delivery system for basic research of adipose biology and gene therapy for adipose-related diseases and beyond.

## Figures and Tables

**Table 1:** Primer sequences used for qPCR.

Gene	Sequence
*Actb*	ACCCGCGAGCACAGCTT ATATCGTCATCCATGGCGAACT
*Adrb3*	GGACGCTGTTCCTTTAAAAGCA TCCATCTCACCCCCCATGT
*Adipoq*	CCCTCCACCCAAGGGAACT CCATTGTGGCCAGGATGTC
*Acaca*	ACGTGCAATCCGATTTGTTGT CCAGCCCACACTGCTTGTA
*Adgre1*	CAGGGCAGGGATCTTGGTTA ACAGGTATTATCAAGGATTGTGAAGGT
*Bdnf*	CCATAAGGACGCGGACTTGT AGGCTCCAAAGGCACTTGACT
*Cfd*	GTGCAAGTGAACGGCACA GTCGTCATCCGTCACTCCATC
*Chrebp1a*	CGACACTCACCCACCTCTTC TTGTTCAGCCGGATCTTGTC
*Cxcl10*	AAGTGCTGCCGTCATTTTCT CTTCCCTATGGCCCTCATTC
*Cd11c*	TGAGAGCCCAGACGAAGACA ACTGAGCTGCCCACGATAAGA
*Fasn*	GATCCTGGAACGAGAACACGAT TGTCAGTAGCCGAGTCAGTCTTG
*Fgf21*	GGAGCTCTCTATGGATCGCCT TGTAACCGTCCTCCAGCAGC
*Gpat*	CAACACCATCCCCGACATC TGACCTTCGATTATGCGATCAT
*Glut4*	TTATTGCAGCGCCTGAGTCT GGGTTCCCCATCGTCAGAG
*Hsl*	GCGCCAGGACTGGAAAGAAT TGAGAACGCTGAGGCTTTGAT
*Il1b*	GCCACCTTTTGACAGTGATGAG GGAAGCAGCCCTTCATCTTTT
*Insr*	GGCTCTCCCCAGGAAACTACA GGTTCTGTCCAGGAGCCATTT
LEP *(human)*	AGACACTGGCAGTCTACCA TGGCAGCTCTTAGAGAAGG
*Lpl*	TCGTCATCGAGAGGATCCGA TGTTTGTCCAGTGTCAGCCA
*Srebp1c*	TGGATTGCACATTTGAAGACATG GGCCCGGGAAGTCACTGT
*Tgfb1*	AGCTGCGCTTGCAGAGATTA AGCCCTGTATTCCGTCTCCT
*Prkce*	ACTGCACCATCCAGTTCGAG GCTCCAGGTCAATCCAGTCC
*Pparg*	ATGGGTGAAACTCTGGGAGATTCA CTTGGAGCTTCAGGTCATATTTGTA
*Ppargc1a*	AAGTGTGGAACTCTCTGGAACTG GGGTTATCTTGGTTGGCTTTATG
*Retn*	AAGAACCTTTCATTTCCCCTCCT GTCCAGCAATTTAAGCCAATGTT
*Scd1*	CGAGGGTTGGTTGTTGATCT GCCCATGTCTCTGGTGTTTT
*Trem2*	ACAGCACCTCCAGGAATCAAG AACTTGCTCAGGAGAACGCA
*Tnfa*	ACGGCATGGATCTCAAAGAC AGATAGCAAATCGGCTGACG
*Ppia*	AATGCTGGACCAAACACAAA CCTTCTTTCACCTTCCCAAA
WPRE	TGGCGTGGTGTGCACTGT GTTCCGCCGTGGCAATA

## Data Availability

The datasets generated and/or analyzed during the current study are available from the corresponding author on reasonable request.
